# An Open Surface Drifter for River Flow Field Characterization

**DOI:** 10.3390/s22249918

**Published:** 2022-12-16

**Authors:** Juan Francisco Fuentes-Pérez, Francisco Javier Sanz-Ronda, Jeffrey A. Tuhtan

**Affiliations:** 1 GEA-Ecohydraulics, Department of Agricultural and Forestry Engineering, University of Valladolid, Campus La Yutera, Avenida de Madrid 44, 34004 Palencia, Spain; 2 Centro Tecnológico Agrario y Agroalimentario, Itagra.ct, Campus La Yutera, Avenida de Madrid 44, 34004 Palencia, Spain; 3 Department of Computer Systems, School of Information Technologies, Tallinn University of Technology, 12616 Tallinn, Estonia

**Keywords:** open hardware, GNSS, GSM, radio communication, rivers, wireless sensor networks, river drifter, river characterization

## Abstract

The continuous observation of flows is required to assess a river’s ecological status, to allocate irrigation withdrawals, to provide sustainable hydropower production and to plan actions as well as develop adaptive management plans. Drifters have the potential of facilitating the monitoring and modeling of river behavior at a fraction of traditional monitoring costs. They are floating objects equipped with sensors able to passively follow the movements of water. During their travel, they collect and transmit information about their movement and their surrounding environment. In this paper, we present and assess a low-cost (<150 EUR) customizable drifter developed with off-the-shelf components. The open drifter is capable of handling the majority of use cases defined in the specialized literature and in addition it offers a general river flow characterization toolkit. One of the main goals of this work is to establish an open hardware and software basis to increase the use of drifters in river studies. Results show that the proposed drifter provides reliable surface velocity estimates when compared to a commercial flow meter, offering a lower cost per data point and in contrast to traditional point measurements it can be used to identify and classify large-scale surface flow patterns. The diverse sensor payload of the open drifter presented in this work makes it a new and unique tool for autonomous river characterization.

## 1. Introduction

### 1.1. Rivers and Humans

Access to freshwater is vital to human life and well-being, thus human settlements have long favored a location close to water bodies and in particular to rivers [1]. Rivers are a fundamental source of primary resources (water, food, or sanitation); they are used as natural highways for transportation and as a source of energy. This relationship has structured and influenced the development of metropolitan areas throughout history [2] and, despite that today the geographical distance to a freshwater source is not as vital for everyday survival [1], our society still depends greatly on freshwater to keep its present lifestyle, whether for irrigation, power generation or to fulfill industrial, domestic, and recreational necessities [3].

Living close to a river has multiple benefits; however, it also can have unwanted consequences for both rivers and populations. On one hand, rivers are subject to artificial alterations of their natural flow regime or their geomorphology, which have detrimental consequences on their ecological functions [4]. On the other hand, living close to a river can make nearby populations vulnerable to increasingly common extreme events of floods [5]. Therefore, modeling and monitoring rivers are crucial to anticipate undesired consequences, not only to ensure the safety of the population but also to develop responsible environmental policies and adaptive management plans.

### 1.2. River Flow Monitoring and Modeling

Monitoring and modeling the behavior of a river is a difficult task, as it depends on a large water system, the basin, which interacts with other factors that can change in space and time, such as vegetation, riverbed conditions, as well as, anthropogenic disturbances. The most common solution to monitor flows in a river requires the installation of fixed monitoring stations. Each of the stations observes and registers the river flow or discharge creating a sensor network of Eulerian (fixed location) observers. This is a common solution and examples include the SAIH network in Spain [6], the BfG network in Germany [7], or the Estonian weather service network [8]. The distribution of monitoring stations is sparse (e.g., one station per 503 km^2^ in Spain [9]), but it is usually sufficient to monitor the global performance of the river network. 

Hydrodynamic models require information including the flow rate, water depth and velocities across a river channel. Once properly calibrated, these models can be used to assess the effects of construction works, improve hydropower generation, flood maps, river restoration, ecological studies or testing and assessment of vulnerabilities to future scenarios. The information provided by existing fixed monitoring stations is usually insufficient due to their sparse spatial distribution. Hydrodynamic modeling is a demanding process [10], which requires precise terrain reconstructions, boundary conditions of each scenario (e.g., discharge and/or water levels), and distributed and referenced data points (velocity and water levels) through an area to calibrate, to assess the model, or to increase the robustness by data assimilation. Therefore, for hydrodynamic modeling, the scattered information provided by the network of gauging stations is usually combined with on-site collected hydrogeomorphological information (point measurements), which in many cases is limited due to the associated collecting costs [11].

### 1.3. Drifters in Hydrology

Water surface drifting buoys have been proposed as a possible tool to facilitate the collection of hydrological information in rivers and riverine environments [12,13,14,15,16]. Water surface drifting buoys (hereafter referred to simply as “drifters”) are floating bodies equipped with sensors and/or communication systems that passively follow the movements of water surfaces, collecting information about their movement and/or their surrounding environment. Drifters function as Lagrangian particles, and have been used for centuries in oceanography [17] to study the global recirculation patterns of the ocean [18,19], to improve weather forecasting [20], to calibrate surface velocity models [19], or to study marine debris pathways [21,22].

The use of drifters in rivers is a re-emerging field of research. In contrast to classical individual point measurements, drifters are able to take into account the Lagrangian nature of the river flow and they allow to measure directly flow patterns rather than trying to infer them from Eulerian or point measurements [11]. In addition, when compared with classical methods (such as flow meters), drifters can autonomously collect more information in less time, which reduces the cost of data collection. 

The use of drifters has produced promising results in river research (Table 1). For instance, [11] shows the use of a GPS-based drifter for flow and velocity characterization in a river, [12] demonstrates the possibility of inferring dispersion and diffusivity of particles in rivers, [16] shows their use for estimating water surface roughness and [14] their capability to improve modeling by using the collected information for data assimilation. Despite the promising published results, drifter technology has not been established as an indispensable tool in the study of rivers.

So far, developed drifters had been used to answer specific research questions (Table 1) and not as a general tool for river characterization or to study multiple problems. The lack of accessibility to this technology is driven primarily by three factors: (1) a lack of available prototype designs [23], (2) low reproducibility in the studied cases, and (3) slow progress in the use and expansion of drifters as a multidisciplinary set of skill is needed for first develop a drifter and then research using it. 

**Table 1:** Summary of relevant drifters for river applications developed during recent years (up to 2020) and their main characteristics. IMU means Inertial Measurement Unit.

Last Seen	Last Reference	Dimensions	Price (EUR)	Positioning	IMU	Real-Time Transmission	Usage
2007	GRiFTers [11]	0.1 m × Ø 0.3 m -kg	140	GPS	No	No	⋅ Flow—velocity mapping
2008	River Drifter [12]	0.46 m × Ø 0.1 m 3.6 kg	≈270	GPS	No	No	⋅ Dispersion and diffusivity studies
2009	QNA [24]	Ø 0.15 m 1.8 kg	>270	GPS	No	Satellite (0.005 Hz)	⋅ Collection of river parameters (depth and velocity)
2015	RTK drifter [25]	0.26 m × Ø 0.2 m -kg	≥€€€€ ^1^	GNSS	No	No ^2^	⋅ Shallow water
2015	Generation 3 [14,26]	0.47 m × Ø 0.13 m >2.8 kg	≈2210	GPS	No	Zigbee and Mobile	⋅ Active (actuated) ⋅ Flow—velocity mapping ⋅ Data assimilation
	Android [14,27]	0.29 m × Ø 0.13 m 2.8 kg	≈440				⋅ Flow—velocity mapping ⋅ Data assimilation
2016	GPS floater [28]	Ø 0.10 m 0.3 kg	≥€€€ ^1^	GPS	No	No	⋅ Dispersion and diffusivity studies
2018	Lagrangian drifter [16]	Ø 0.03 m 9 × 10^−3^ kg	≥€€ ^1^	No	9 DOF	No	⋅ Water surface roughness studies
2019	Low power drifter [23]	-	125	GPS	3 DOF	Mobile (0.017 Hz)	⋅ Drifter location
2022	Open Drifter (this work)	0.07 m × 0.1 m × 0.05 m 0.35 kg	<150	GNSS	9 DOF	LoRa radio or Mobile (1–2 Hz) ^2^	⋅ Flow—velocity mapping ⋅ General river characterization (velocity mapping, orientation, forces, angular rate, etc.)

^1^ Estimated price range considering the components used. ^2^ Onboard storage up to 10 Hz.

Drifters have substantial potential to change how rivers are measured and understood, however, without a common starting point among potential users of this technology, it is not possible to advance the techniques or to reduce its application costs. 

### 1.4. Purpose of This Paper

This paper aims to establish an open hardware and software basis (https://www.gea-ecohidraulica.org/GEA_en/sensors/drifter.html (accessed on 14 December 2022)) to make possible the application of drifter technology to potential users (e.g., river researchers or engineers), without a highly technical background to develop a custom drifter and to provide a common and reproducible reference point for the research and applications. To achieve this, we have developed a new customizable drifter with commercially available, low-cost (<150 EUR with the maximum number of components) and off-the-shelf components. The new open drifter satisfies the functional hardware requirements to handle and merge all study cases listed in Table 1 due to multiple sensing modules, communication technologies, and high sampling frequency (up to 10 Hz on board). In addition, the provided open software interface allows the near real-time data visualization of the deployed drifters via mobile or radio connectivity (for rivers outside mobile coverage). 

In addition, to study and show the performance of the developed drifter, a characterization of a reach of the Pirita River (Estonia) has been carried out. The study shows the pros and cons of drifters as well as their potential to improve rivers’ monitoring, characterization, and understanding. In the same way, clear future steps are listed to continue the evolution and to exploit all the potential of this technology. 

The paper is organized as follows: Section 2 describes the materials and methods used in to construct the drifter: the hardware architecture (Section 2.1), software architecture (Section 2.2), the experimental setup (Section 2.3) and the data structure and treatment (Section 2.4). Section 3 provides the results from a field study and a discussion of its performance: an overview of the drifter after assembly (Section 3.1), method to estimate the surface and river current velocity (Section 3.2) and river characterization based on surface flow observations from the drifter (Section 3.3). Finally, Section 4 summarizes the overall findings, conclusions and future perspectives.

## 2. Materials and Methods

To develop a suitable river drifter technology, we started by analyzing the current state of the art and their applications for river research and identified the main limitations. By doing so, we composed a list of design characteristics:

1. It needs to track its location, velocity, and orientation precisely.
2. It must be able to transmit its state/data in real-time, with and without mobile connectivity.
3. It needs to have a software platform to visualize its state.
4. It must be easily configurable in terms of buoyancy, shape, and hardware.
5. It must be designed to be open and use mainly open technology.
6. It must be capable of storing data at a sampling rate higher than 1 Hz (faster than most previously published devices).
7. It must be able to operate for at least 1 day continuously without losing power.
8. It must be able to handle sleeping mode, for low sampling rates and longtime measuring scenarios.
9. It must be a low-cost technology (< 200 €).

Considering the list of desired characteristics, the design was divided into three main tasks: (i) component selection, design of the device and low-level microcontroller programming (Section 2.1), (ii) programming of the real-time state visualization software (Section 2.2) and (iii) real test of the technology (Section 2.3 and Section 2.4).

### 2.1. Hardware

**Figure 1:**
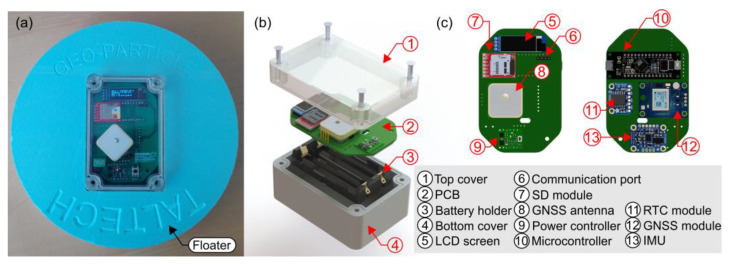
The open drifter and its basic components. (**a**) Drifter installed in an example float. (**b**) Basic hardware components. (**c**) Data logging module with all the components.

The drifter here presented has been completely designed with off-the-shelf modules connected to each other with a custom printed circuit board (Figure 1). The complete documentation (software and hardware) for its replication is openly available at https://www.gea-ecohidraulica.org/GEA_en/sensors/drifter.html (accessed on 14 December 2022). Due to the selected housing (an IP68 box), the current form factor is 70 mm × 102 mm × 52 mm (0.35 kg), inserted in a 20 cm floater (shallow water mode). The dimensions of the housing and floater can be easily modifiable according to the requirements of the rivers where the drifter is going to be deployed. In the most expensive configuration, i.e., with the maximum amount of components, the total material cost of the drifter is lower than 150€. This price can be lowered by removing components not needed for a specific use case.

The drifter consists of two main components, each one with its own microcontroller: (1) the sensing and onboard data logging module, which manages the data collection and storage; and (2) the data transmission module, responsible for wireless data communication.

#### 2.1.1. Data Logging Module

**Figure 2:**
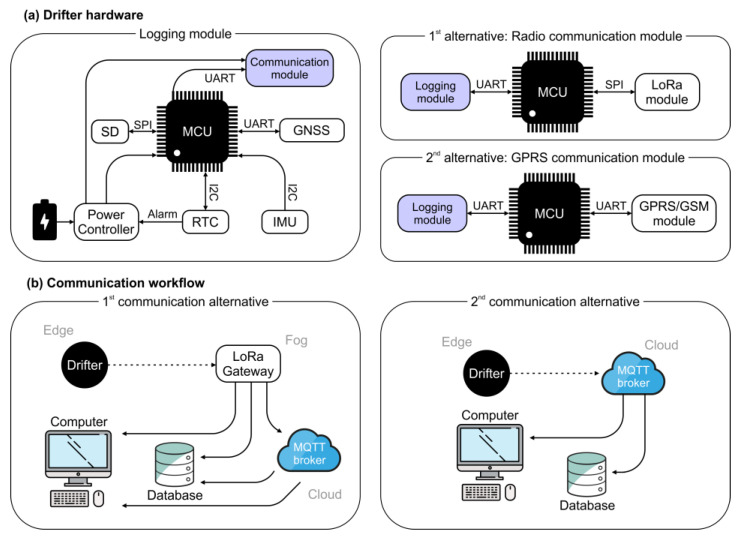
Overview of the open drifter hardware and communication architecture. (**a**) Hardware and communication architecture of the logging and communication modules. (**b**) Communication workflow alternatives between a drifter and near real-time visualization.

The data logging module is the main module of the drifter. Its main components consist of an Arduino compatible [29] M0 microcontroller board, connected to a NEO-M8T Global Navigation Satellite System (GNSS) board [30], a BNO005 Inertial Measurement Unit (IMU) board [31], a DS3231 Real Time Clock (RTC) board [32] and a Pololu power switch [33]. The module is powered by 3.7 V 18650 Li-Ion batteries and it logs the data to an SD card [34] at 5 to 10 Hz in ASCII text file format. Figure 2a summarizes the components and connections of the board and modules. 

#### 2.1.2. Data Transmission Module

The data transmission module is based on an Arduino compatible M0 microcontroller board and offers two possible modes of communication: (i) radio or (ii) Global System for Mobile Communication (GSM) (Figure 2a). The GSM is performed by a SIM800L board [35] and radio via a RFM95 LoRa radio board. In both cases, the microcontroller is subscribed to the data logging module via serial communication. This provides for data logging independently from the communication process. The data communication is transmitted at a rate of 1 Hz, using a MQTT (Message Queue Telemetry Transport) online broker in the case of GSM, or using a gateway to transmit the information directly to a computer or a MQTT broker in the case of radio (Figure 2b). The two options are made available as many use cases are remote and do not afford reliable cellular communication access using a GSM transceiver.

### 2.2. Software

**Figure 3:**
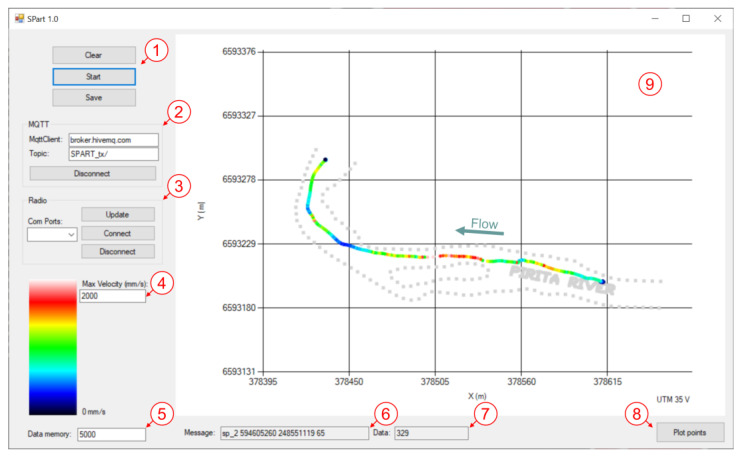
SPart software for drifter tracking with different communication alternatives. The figure shows the track of a drifter with mobile communication in the studied reach. (1) Main control menu. (2) Connection to MQTT broker. (3) Connection to Radio Gateway. (4) Velocity scale definition. (5) Number of positions on the screen. (6) Latest drifter message received. (7) The total number of messages received. (8) Load a set of reference points (gray point background). (9) Map with the received data and positions (automatically converts data from WGS84 geographical coordinates to UTM format for representation).

The drifter’s real-time data is visualized using the open software SPart (Figure 3), a custom-made software developed using Windows Forms and C-sharp in Visual Studio 2019. This software (openly accessible at https://www.gea-ecohidraulica.org/GEA_en/sensors/drifter.html (accessed on 14 December 2022)) is able to handle data transmitted from a MQTT server as well as serial data provided by a LoRa radio gateway, both separately and together. Thus, it is usable by both defined transmission modalities. The intuitive and user-friendly interface of SPart allows to plot in real-time the position of the different drifters deployed together with their velocity. This allows a rapid assessment of the collected data, to make deployment decisions, and to localize the deployed drifters at any moment. Additional data processing techniques, such as filtering or data assimilation, have not been implemented yet in the software. 

### 2.3. Study Case and Experimental Setup

**Figure 4:**
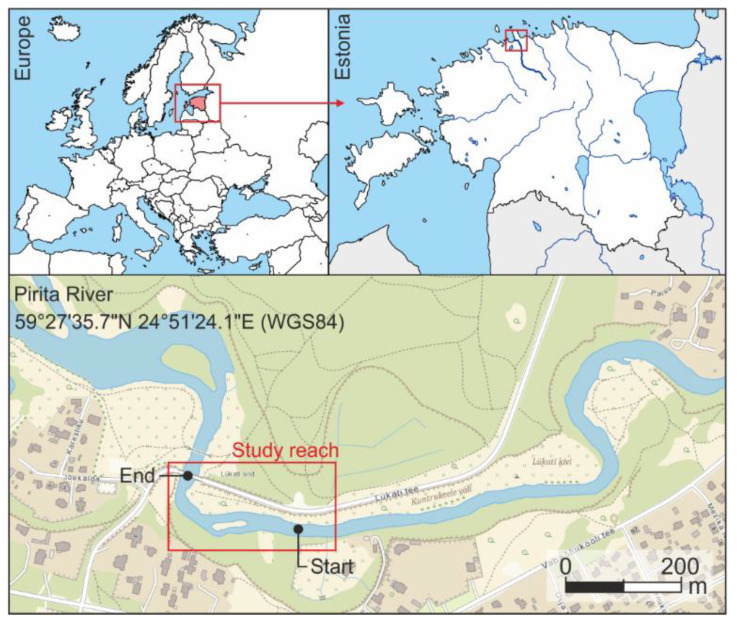
The situation of the study reach. Pirita River, Estonia.

Field trials were conducted in the Pirita River (Figure 4), a 105 km long river in northern Estonia that empties into the Baltic Sea. The study reach has a length of around 300 m, with diverse hydro-morphological units (e.g., pools, riffles, or rapids). The mean water depth in the reach during the field test was mainly lower than 1 m (shallow water). Tests were conducted on the 30 May 2019, in Kloostrimetsa gauging station [8] the water level was 106 cm, the water temperature: 15.8 °C, and the air temperature: 16.6 °C. 

Two experiments were performed, and three identical drifters were used during all experiments: The first experiment was designed to compare the velocities registered by drifters with a standard propeller-type current meter: Geopacks Advanced Stream FlowMeter. For this, in the first place, four locations with different velocities were selected visually in the river reach. After that, 10 repeated measurements of the velocity in each location were registered with the flow meter at 40% from the riverbed (mean velocities of 0.30 m/s (depth = 78 cm), 0.69 m/s (depth = 83 cm), 0.86 m/s (depth = 80 cm), and 1.40 m/s (depth = 66 cm). To compare those velocities with the drifter velocity estimates, in each location a 4 m long track was defined. Each track was recorded five to sixteen times with the drifters (n_1_ = 16, n_2_ = 15, n_3_ = 5, and n_4_ =15).

The second experiment consisted of the characterization of the selected reach to integrate the information of the drifters into a single map of the reach. For this, nine deployments using three drifters in the same deployment position were carried out, collecting them at the end of the study reach (Figure 4). 

### 2.4. Data Structure and Treatment

Table 2 summarizes the recorded data with the drifters at a sampling rate of 5 Hz during the performed tests. The number of variables can be increased or reduced by modifying the code of the logging module microcontroller. 

For the first test, to compare the velocity measured by the drifters with the current meter velocity, a single mean velocity value was calculated from each track of the drifter, removing the beginning (drifter acceleration when released) and the end (recovery influence) through visual inspection. After that, data collected with the drifter and current meter for each location were compared, using non-parametric Mann-Whitney U-test (ranksum), and evaluating the correlation between measurements in all locations (Spearman correlation coefficient, corr). All statistical analyses and post-processing were performed using Matlab R2019a. 

Data from the second test (reach characterization) were integrated into a single map, transforming the tracks of the drifters to a 2 m by 2 m spatially fixed cell map. For this, only data with horizontal accuracies lower than 2 m were considered (observed mean accuracy estimation = 0.75 m). After that, with the data of the nine different tracks inside a cell, the mean value of recorded variables, number of data points, and standard deviation of each cell were calculated. 

## 3. Results and Discussion

### 3.1. About the Drifter and the Open Platform

The hardware and sampling rate of the proposed drifter provide the necessary specs to perform most of the tasks defined in the specialist literature (Table 1). Moreover, the combination of the multiple sensing modules in the drifters allows to go beyond those tasks by combining the multiple variables recorded. 

Regarding the position tracking technology, a module able to connect to multiple Global Navigation Satellite Systems (GNSS) was selected. Although it is the most expensive module in the drifter, it has multiple benefits. The selected module (NEO-M8T) is compatible with GPS, GLONASS, BeiDou, QZSS, SBAS, and Galileo satellite systems and it can combine their information, ensuring sufficient coverage and precision in remote areas. In addition, it has already navigation solutions (e.g., ground speed, heading, etc.) built on-board, which simplifies the data processing. 

Aware of the limitations of mobile coverage in many of the potential study sides, two communication alternatives were implemented: mobile and radio. Both alternatives have been tested and it is possible to use drifters with radio at the same time as drifters with mobile connectivity, tracking them simultaneously using the Spart software. Their main differences are the power consumption (mobile > radio) and the data transmission workflow (Figure 2). Mobile communication allows receiving real-time data in any part of the world, making easier office data processing while performing field studies. However, the needed coverage and associated costs (mobile operator) have favored conducting most of our tests using radio.

One of the most interesting features of the drifter is the IMU sensor. The selected module (BNO055) is a 9-DOF sensor, which includes a triaxial accelerometer, a triaxial gyroscope, a triaxial geomagnetic sensor, and its microcontroller that performs sensor fusion on its own, providing not only individual values of each sensor but also an estimate of the absolute orientation. The possible applications of this technology for river characterization are still an emerging but promising line of research [16,36]. The fusion of GNSS data with IMU data can change the way of analyzing and understanding river systems. 

Together with the drifter, we developed a visualization tool to track the drifters over the study reach and for rapid data assessment (Figure 3). We found this tool vital for field applications to not only ensure that the sensor coverage was enough and correct to perform the characterization of rivers but also to find and retrieve missing drifters. 

All drifter prototype designs, code, and software are openly available at https://www.gea-ecohidraulica.org/GEA_en/sensors/drifter.html (accessed on 14 December 2022), intending to extend the usage of this technology in different study cases, to accelerate its evolution and to provide a common starting point to all interested people. 

### 3.2. Comparison with the Propeller-Type Current Meter

**Figure 5:**
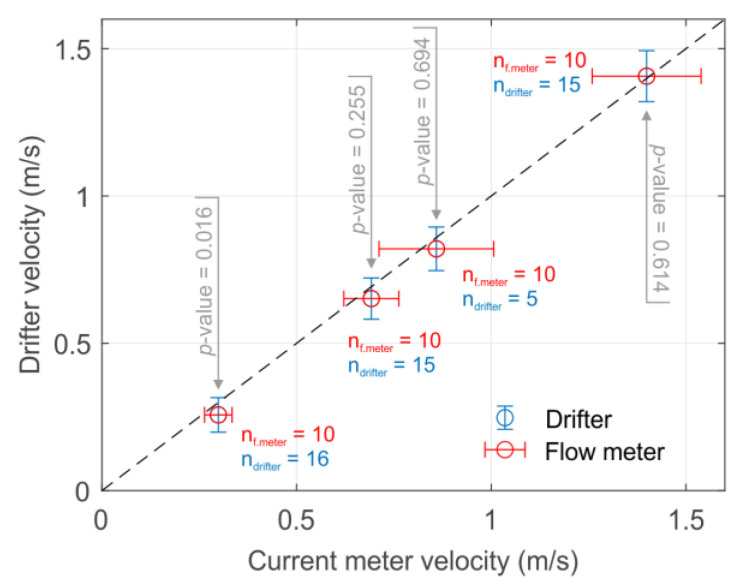
Comparison of the velocity estimated by a current meter and the drifter. Only for the lowest velocity a significant difference in the measurements is observed.

The comparison between current meter measurements and drifter’s velocity estimates only showed significant differences (α = 0.05) in the lowest velocity recorded (Figure 5). Measurements showed a strong correlation between them (ρ = 1). In general, it was found that the drifter measurements had a lower variance than the current meter, which can be explained by the fact that drifter velocity is estimated using a higher number of values per sample. The drifter data processing acts as a mean filter when calculating the velocity value, reducing the variance in each location. This is similar to [11], which reported in their tests that the larger number of values to estimate the velocity the lower the standard deviation. However, this comes at a reduced ability to resolve small-scale variations in velocity. In addition, differences between the current meter and drifter measurement were found to decrease with increasing velocity. This can be related to the design of the first test, as the drifters started with a velocity of 0 m/s in each deployment, and slowly accelerated until they reached the real flow velocity. Therefore, despite eliminating part of the beginning of the track, lower velocities will require longer times and distances to reach the real flow velocity and thus, the relative error committed in the estimate can be higher. This is an important consideration, as any obstacle that interacts with the drifter may decelerate it, spreading a small error in the first meters downstream of the obstacle. This can be solved (1) by identifying and removing such samples in the recorded track or (2) by increasing the number of data points using multiple particles and tracking the standard deviations, anomalies, and the number of samples in any location. 

In general, slightly higher estimates of the velocity were expected in the drifter due to the velocity profile in open channel flows [37], which only happens marginally in the highest velocity measured (Figure 5), probably due to a low roughness that generated a homogeneous velocity profile in the selected points. The experimental design may explain this issue, but also the selected floater could have influenced it. The used floater provides a high buoyancy that makes the drifter compatible with low-depth areas (<10 cm in some locations); however, it also provides greater exposure to the wind. This exposure could also influence the measurements [21], revealing a similar pattern to the observed one, where the relative deviations would have been more obvious for lower velocities. An optimized floater with lower buoyancy can aid in reducing the wind influence (i.e., lowering the drag area). This is the reason a modular design was selected, to be able to adapt the drifter to different rivers and environmental conditions. In an ideal case, different drifter shapes could be used to characterize different areas of the river. 

Considering the above and due to the observed few differences, we cannot draw any sound conclusion, we can just point out possible limitations and solutions, but we can validate the technique as well as the study case for deeper characterization. 

### 3.3. River Reach Characterization

The use of drifters has some limitations that must be considered and controlled for its correct use. However, it offers many advantages for river characterization when compared with classical point measurement methods. First, it can provide rapidly numerous measurements in a single release reducing the human and time cost; moreover, it can provide flow measurement in extreme scenarios (e.g., flood conditions or rapids) and inaccessible reaches. In addition, due to its Lagrangian nature, it provides continuous measurements allowing the identification of flow patterns, adding another exploitable dimension to the flow characterization of rivers and useful to understand better the river performance or to validate and complement CFD model [14]. 

**Figure 6:**
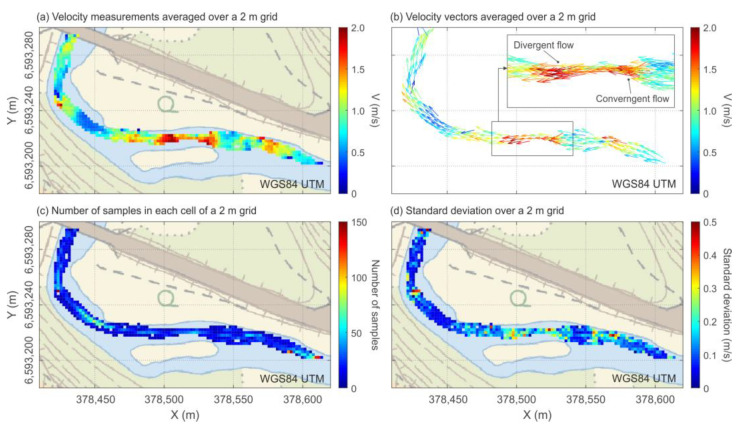
Integration of the nine independent tracks of the drifters. (**a**) Velocity measurements averaged over a 2 m by 2 m rectangular cell grid. (**b**) Velocity vectors averaged over a 2 m by 2 m rectangular cell grid. (**c**) Number of samples in every 2 m by 2 m rectangular cell grid. (**d**) Standard deviation in every 2 m by 2 m rectangular cell grid.

Figure 6 summarizes the integration of the nine independent releases of the drifter in the studied reach. Figure 6a shows the Eulerian conversion of the drifter tracks in 2 by 2 m cells surface velocity map. The technique provides spatially distributed continuous measurements that range from 0 in pool areas to 2.2 m/s in rapids. The vector plot (Figure 6b) clearly shows areas of convergent flow upstream of the rapids and divergent flow downstream. Similar observations were made in [11,12]. These features and observations are beyond the usage range of classical methods. Moreover, a higher resolution (thinner cells) or the study of individual paths of the drifters could allow the characterization of more complex features of the flow (e.g., eddies) or even localize obstacles to the flow (e.g., rocks). 

In addition, it is possible to perform a rapid assessment of the collected data by plotting the number of data samples used to calculate each value of 2 m by 2 m cells (Figure 6c) as well as their standard deviation (Figure 6d). Due to the direct information provided, it is possible to implement a workflow for rapid data assessment on the field. This allows the user to concentrate the sampling effort on those areas with a lower density of observations needed for a robust characterization. Figure 6c illustrates that it is not uncommon to find highly repeatable pathways followed by the drifters. These result in areas with a higher probability of presence. By changing the release locations, it is possible to cover areas with low spatial coverage in previous deployments. In addition, in the study case, a higher standard deviation is observed in areas with high velocities (Figure 6d). Those areas are in general more complex (i.e., high diversity of hydraulic conditions per spatial unit) and the standard deviations tend to increase with an increase in the grid size. This can be solved similarly to CFD models, by adapting a range of grid sizes in the reach after a preliminary characterization, that is to say, a smaller grid for more complex areas while a larger grid for more homogenous areas. 

**Figure 7:**
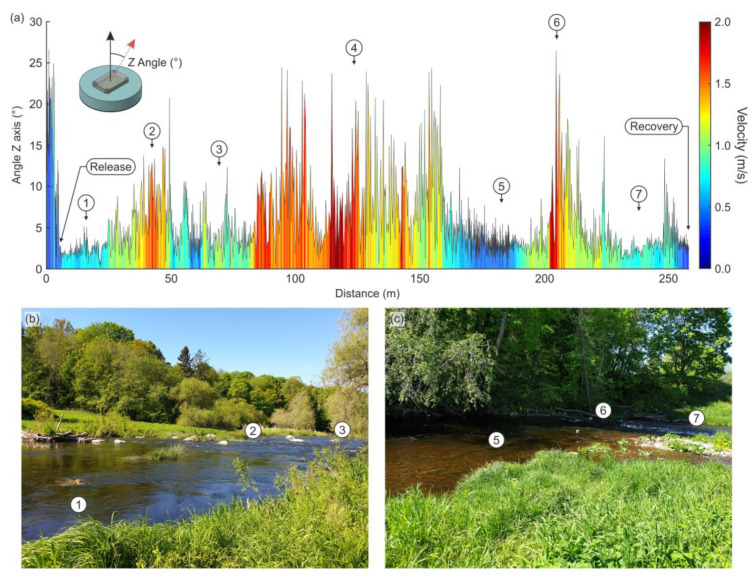
The inclination of the drifter during the studied track (raw data). (**a**) Evolution of the velocity and inclination during the track. This allows the differentiation of areas of different properties within the reach; (**b**,**c**) different areas and transitions. Numbers link the graph (**a**) with the different river arear (**b**,**c**).

Apart from river flow and velocity characterization, the set of variables provided by the IMU allows the characterization of the reach beyond the previous works performed with drifters [11,12,13,14]. The conjunction of the different features recorded provides a unique and reproducible signature of the studied reaches. As an example (aware of the multiple possible combinations of variables) in Figure 7 we represent the inclination of the drifters (directly related to water surface roughness and turbulence [16]) together with the velocity during its travel through the reach. This figure indicates that drifters could be useful for the detection, characterization and classification of different hydrogeomorphological units in the reach [38], by providing a simple and fast method for large-scale surveys of hydrodynamics and investigating physical and biological processes in natural rivers and streams. Of course, this and similar methods or variable combinations, as well as the automation of the classification process needs to be further investigated, tested, and validated considering classical methods, as it goes beyond the objectives of this work. 

## 4. Summary and Conclusions

The developed surface drifter offers a new, open access and inexpensive platform to measure and monitor river surface flows. Moreover, due to the combination of the onboard sensors, new research opportunities have arisen related to river classification. 

In general, geolocated drifters provide several advantages over traditional Eulerian fixed station observations, but there are limitations requiring further consideration for their use. When compared with traditional methods, drifters provide only the surface velocity. Despite the existence of methods for transforming surface velocity to mean velocity in well-known channel shapes, further study and analysis are required for its application in natural channels. Adding an echosounder for water depth measurement in combination with other sensors already available, would give interesting information to achieve this task [24], but at a higher cost. As an example, the Ping sonar from BlueRobotics [39] would be compatible with the design at an increase in the cost of 342 EUR. 

The most important factors that have limited the usage of this technology to date are the absence of specific commercial alternatives and a big user community behind them. This has been one of the main motivations for the present paper, to provide access to potential users to an open device and a set of tools, which are currently under development and testing in different river characterization problems. 

Our future work is focused on the application of this technology to more complex problems (among others: the study of glacial hydrology [36,40], the characterization of hydraulic structures, the analysis of velocity barriers for fish, or the evaluation of the performance of fishways [41,42]). Regarding the design, we are carrying out a serial study of the effect of drifter shape in its performance as well as including other sensors to the platform (such as depth sounders or water quality sensors). Our hope is that river drifters will become a highly-reliable and low-cost measurement system for the study of river flows. 

## Figures and Tables

**Table 2:** Summary of the data recorded by the drifters and units (UTC = Coordinated Universal Time; WGS = World Geodetic System; NED = North-East-Down system).

Column	Variables		Units
**Microcontroller**			
1	Time		ms
**GNSS**			
2	Time		ms
3	UTC Time		ms
4	Nano UTC time		ns
5	Latitude (WGS84)		10^−7^ degrees
6	Longitude (WGS84)		10^−7^ degrees
7	Height above ellipsoid		mm
8	Horizontal accuracy		mm
9	Ground speed		mm/s
10	NED north velocity		mm/s
11	NED east velocity		mm/s
12	NED down velocity		mm/s
13	Number of satellites		-
14	Heading		10^−5^ degrees
**IMU**			
15	Quaternions	w	-
16		y	-
17		x	-
18		z	-
19	Acceleration	x	m/s^2^
20		y	m/s^2^
21		z	m/s^2^
22	Magnetic field	x	µT
23		y	µT
24		z	µT
25	Gyroscope	x	rad/s
26		y	rad/s
27		z	rad/s
28	Calibration of gyroscope		-
29	Calibration of accelerometer		-
30	Calibration of magnetometer		-

## Data Availability

Data are available upon reasonable request to the corresponding author.
